# Nowcasting the Spread of Chikungunya Virus in the Americas

**DOI:** 10.1371/journal.pone.0104915

**Published:** 2014-08-11

**Authors:** Michael A. Johansson, Ann M. Powers, Nicki Pesik, Nicole J. Cohen, J. Erin Staples

**Affiliations:** 1 Division of Vector-Borne Diseases, Centers for Diseases Control and Prevention, San Juan, PR; 2 Division of Vector-Borne Diseases, Centers for Diseases Control and Prevention, Fort Collins, Colorado, United States of America; 3 Division of Global Migration and Quarantine, Centers for Diseases Control and Prevention, Atlanta, Georgia, United States of America; Singapore Immunology Network, Agency for Science, Technology and Research (A*STAR), Singapore

## Abstract

**Background:**

In December 2013, the first locally-acquired chikungunya virus (CHIKV) infections in the Americas were reported in the Caribbean. As of May 16, 55,992 cases had been reported and the outbreak was still spreading. Identification of newly affected locations is paramount to intervention activities, but challenging due to limitations of current data on the outbreak and on CHIKV transmission. We developed models to make probabilistic predictions of spread based on current data considering these limitations.

**Methods and Findings:**

Branching process models capturing travel patterns, local infection prevalence, climate dependent transmission factors, and associated uncertainty estimates were developed to predict probable locations for the arrival of CHIKV-infected travelers and for the initiation of local transmission. Many international cities and areas close to where transmission has already occurred were likely to have received infected travelers. Of the ten locations predicted to be the most likely locations for introduced CHIKV transmission in the first four months of the outbreak, eight had reported local cases by the end of April. Eight additional locations were likely to have had introduction leading to local transmission in April, but with substantial uncertainty.

**Conclusions:**

Branching process models can characterize the risk of CHIKV introduction and spread during the ongoing outbreak. Local transmission of CHIKV is currently likely in several Caribbean locations and possible, though uncertain, for other locations in the continental United States, Central America, and South America. This modeling framework may also be useful for other outbreaks where the risk of pathogen spread over heterogeneous transportation networks must be rapidly assessed on the basis of limited information.

## Introduction

In December 2013, the first locally-acquired chikungunya virus (CHIKV) infections in the Americas were reported from St. Martin [1]. CHIKV is transmitted to humans by *Aedes aegypti* and *Ae. albopictus* mosquitoes and can cause explosive outbreaks of fever and severe polyarthralgia affecting 30–75% of the population [2], [3], [4]. Prior to 2013, outbreaks of chikungunya had been reported in Africa, Asia, Europe, and islands in the Indian and Pacific Oceans. While CHIKV transmission had never been documented in the Americas, the potential for outbreaks had long been recognized because of the prevalence of the vectors and their efficiency at transmitting dengue viruses [5].

As of May 16, 55,992 locally acquired and travel-related cases had been reported from fourteen islands in the Caribbean and French Guiana [6]. Although further spread is probable, the current extent of spread and risk is uncertain. Uncertainty arises from numerous factors including challenges in assessing the current prevalence of infection and travel patterns, the complexity of the transmission cycle, and stochasticity in outbreak propagation. Measuring the prevalence of CHIKV is challenging as cases might be unrecognized, confused with other diseases such as dengue, or not reported. Travel patterns are also difficult to capture in real-time and might change due to the outbreak itself. Transmission potential is difficult to predict due to differences in mosquito species, vector competence, and vector densities [7], [8], [9], [10], [11], [12], [13]. Lastly, epidemics are inherently stochastic; there may be numerous possible routes of spread, but by chance only some will actual occur. Given the many unknown entities, models considering both the available data and the associated uncertainty can provide insight on the most probable routes of spread and the locations where unrecognized cases may already be occurring.

To estimate the current risk of CHIKV spread, we utilized two branching process models [14]. The first model estimates the probability of at least one CHIKV infected traveler arriving somewhere as a single step process dependent on (1) the number of infected individuals in locations where transmission has occurred, (2) the probability of those individuals travelling, and (3) the duration of infection. The second model estimates the probability of CHIKV transmission spreading to new locations as a three-step process: (1) an infected traveler must arrive; (2) that traveler must infect at least one mosquito; and (3) at least one infected mosquito must infect at least one person. We incorporated uncertainties into these models using global sensitivity analysis and predicted the probability of infected travelers and the initiation of autochthonous transmission for each of the first five months of the outbreak (December 2013–April 2014).

## Methods

### Models

Based on previous work [14], we estimated the probability of an infected traveler arriving in location (*i*) as a binomial process dependent on the number of infections (*I*) in each source location (*s* in *S*) in each month (*m* in *M*), the average duration of infection in humans (*D*), and the monthly probability of travel from each source location (*p_i,s,m_*):
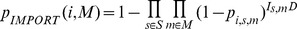



Next, we considered the specific components of DENV transmission from humans to mosquitoes and from mosquitoes to humans [15]. We characterized each of these as a Poisson process with means 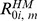 and 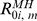, the average number of infectious mosquitoes produced per infected human and the average number of humans infected per infectious mosquito, respectively. 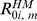 is the product of the number of mosquitoes per person (*φ*), the daily biting rate (*α*), the probability of transmission given an infectious blood meal (*β_HM_*), the number of days a human is infectious (*V*), and the proportion of mosquitoes surviving the extrinsic incubation period (*γ*):
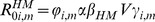




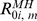
 is the product of the daily biting rate (*α*), the probability of transmission given an infectious bite (*β_MH_*), and the number of days an infectious mosquito survives (*L*):
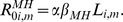



We used these to estimate the probability of introduction leading to autochthonous transmission as the probability of infected travelers arriving, infected travelers infecting mosquitoes, and infected mosquitoes infecting at least one human:
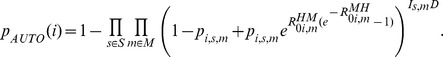



The parameters are described in detail below. Since some parameters (*L*, *φ*, and *γ*) vary with temperature, we used average monthly temperature data for the years 1993–2012 from the NOAA/NCEP Reanalysis dataset (www.esrl.noaa.gov/psd/data/reanalysis) [16] to estimate location- and month-specific parameters. To account for uncertainty in each parameter, we sampled 10,000 sets of parameters from likely ranges of each. For each location we estimated *p_IMPORT_* and *p_AUTO_* with all 10,000 parameter sets, reporting the mean and the 2.5^th^ and 97.5^th^ percentiles of their distributions. Figure S1 in File S1 shows the influence of this uncertainty and temperature on the predicted range of *R*_0_, the basic reproduction ratio. Estimated *R*_0_ peaked at 5.2 at approximately 29°C, with 50% of the values between 1.7 and 6.5.

### Probability of travel (*p*)

We collected data on all itineraries originating from locations with documented CHIKV infections for the period December 2012–April 2013 from Data In, Intelligence Out (www.diio.net). We calculated an initial origin-destination-specific probability of travel for each month as the total number of daily travelers for each origin-destination pair divided by the population of the origin location (an island or metropolitan area). To estimate travel for infected individuals in the months of December 2013–April 2014, we reduced these probabilities by 25–100% (uniformly distributed, mean = 62.5%). This reduction was used to reflect possible changes in travel patterns or differences in the probability of travel for infected individuals due to different risks (e.g., higher risk of infection for non-travelers vs. travelers) or due to illness (i.e., sick individuals may be less likely to travel).

### Infections in source populations (*I*)

We collected data on the reported numbers of suspected and confirmed cases from the Pan American Health Organization and the French Institute for Public Health Surveillance for locations with local transmission reported by the end of April 2014 [6], [17], [18], [19], [20], [21], [22], [23]. These locations were: Anguilla, Antigua, the British Virgin Islands, Cayenne, Dominica, the Dominican Republic, Guadeloupe, Martinique, St. Barthelemy, St. Martin, St. Kitts & Nevis, and St. Vincent & Grenadines. Approximately 80% of CHIKV infected individuals have symptomatic infection with fever and arthralgia and can be identified as chikungunya cases [3], [24], [25], [26]. However, cases may be under-recognized, under-reported, and misclassified (e.g., dengue cases misdiagnosed as chikungunya or vice versa). We estimated that the reported cases represent approximately 80% (standard deviation [SD] 10%) of all infections.

### Human infectious period (*V*)

The human infectious period was considered as the time when infected humans could infect mosquitoes with CHIKV. The level of viremia in humans that is infectious to mosquitoes varies across strains of virus and species and strains of mosquito, with viremia on the order of 10^4–6^ plaque forming units/ml being infectious [7], [8], [9], [10], [11], [12], [13]. Chikungunya viremia above 10^4–5^ typically lasts 3–4 days, post-disease onset [27], [28], [29], [30]. Little data exists on CHIKV infection prior to symptom onset, but humans are likely infectious for 1–2 days before becoming ill [31]. We estimated that the average human infectious period was 4–6 days, or 5 days (SD 1 day).

### Duration of infection in humans (*D*)

We define this period as the length of time between when a human becomes infected and when that human ceases to be infectious to mosquitoes, i.e. the period when a person could travel and still be infectious after traveling. The mean intrinsic incubation period for CHIKV is approximately 3 days [32] and the infectious period post-onset is 3–4 days (above). *D* is thus 6.5 days (SD 1 day).

### Mosquito biting rate (*α*)

A detailed study of blood meals suggests that *Ae. aegypti* feed 0.63–0.76 times per day [33]. We assumed that *Ae. albopictus* behaves similarly and used a mean of 0.7 blood meals per day (SD 0.05).

### Human-to-mosquito transmissibility (*β_HM_*)

*β_HM_* is the probability of a mosquito acquiring CHIKV while feeding on an infectious human. Because we estimated the human infectious period based on the 50% infectious dose, we assume that *β_HM_* is 0.5 (SD 0.1).

### Extrinsic incubation period (EIP)

EIP is the period in the mosquito after acquiring the virus and prior to being able to transmit the virus. This differs by species and strain [8], [9], level of host viremia [8], and most likely by temperature [34]. EIP can be as little as 2 days with a high virus titer blood meal [10], [11], but the average is more likely 4–5 days for efficient vectors with high titer blood meals and 7 or more days for less efficient vectors with low titer blood meals. Temperature-specific data for CHIKV are limited to the range of 26–30°C. We assumed that average EIP at 28°C (*EIP*_28_) was 6 days (SD 2 days) and that the relationship with temperature was similar to that of dengue viruses, *β_T_* = −0.08 (SD 0.02) [34]. We sampled from both distributions to estimate the mean EIP for each location as a function of temperature using the following equation: 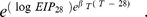


### Mosquito survival (*γ* and *L*)

*Aedes* mortality in the field depends on many factors including weather and species [35]. We assumed that species composition of *Aedes* is unknown and estimated mean mortality for *Ae. albopictus* and *Ae. aegypti* across temperature by averaging mortality for each species at each temperature [35] and fitting a polynomial curve to the relationship between temperature and average daily mortality: 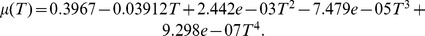 We assumed that the month- and location-specific average mosquito lifespan (*L*) was 1/*µ*(*T*) days (SD 2 days). The proportion of mosquitoes surviving the EIP (*γ*), was then calculated as 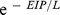, thus incorporating the uncertainty associated with both mosquito mortality and the EIP (above).

### Mosquito density (*φ*)

We assumed that under ideal weather conditions there are 1–3 mosquitoes per person, an average of 2 (SD 1). To account for the population-wide effects of increased mortality at temperature extremes, we estimate the density proportional to the minimum mortality 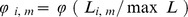, where *φ* is the density under ideal weather conditions, *L_i,m_* is a location- and month-specific, temperature-dependent lifespan, and max *L* is the maximum mean lifespan, 7.9 days.

### Mosquito-to-human transmissibility (*β_MH_*)

Transmissibility of CHIKV from infected mosquitoes to humans is unknown, yet it is likely less than 100%. We assumed that the probability was 0.5 (SD 0.1).

## Results

### Probability of chikungunya virus infections among travelers

**Figure 1:**
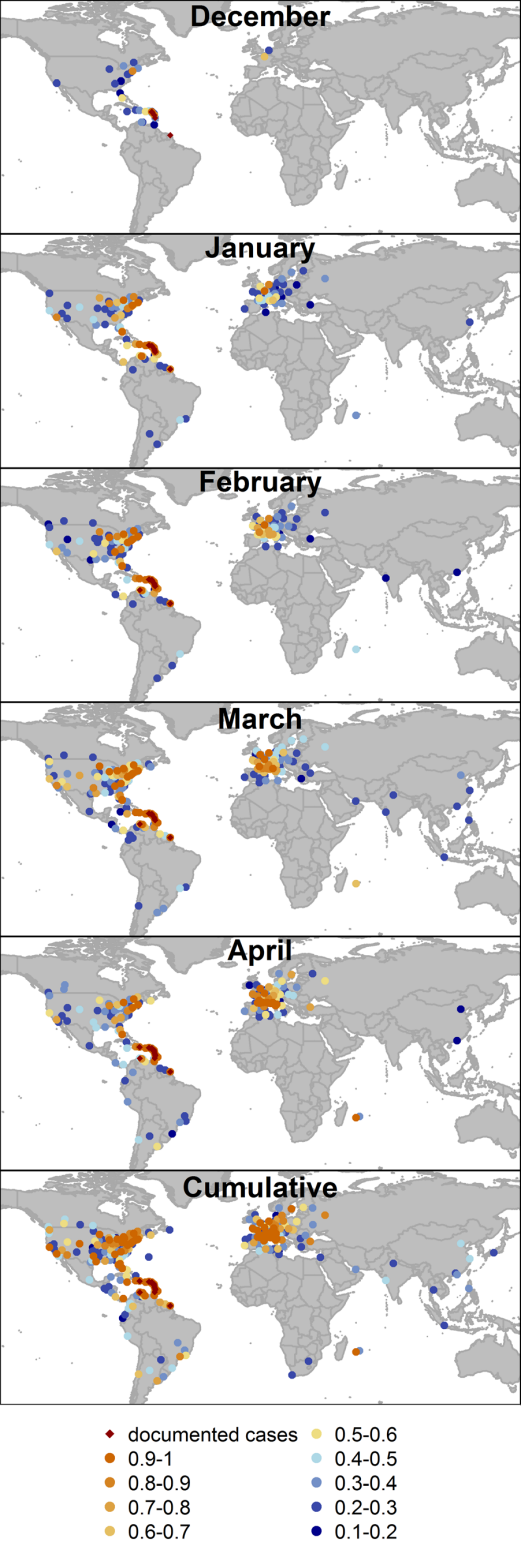
Probability of chikungunya virus importation by location, December 2013–April 2014. Location-specific predictions of the probability of the arrival of at least one chikungunya infected traveler by month and cumulatively over the 5-month period. Locations shown have mean probabilities of importation greater than 0.1. Locations which had reported locally-acquired or travel-related cases in that month or previous months are marked in red.

As the outbreak has evolved in the Caribbean, the predicted risk of CHIKV infected travelers arriving in other locations around the world has generally increased on a monthly basis (Figure 1). In December, only 5 locations had a probability greater than 0.5 of having an infected traveler arrive. Over the next four months of the outbreak, the number increased to 40 in January, 57 in February, 82 in March, and 65 in April. The slight decrease in April reflects the lower number of new cases reported from St. Martin and St. Barthelemy. All locations that had documented imported or autochthonous cases as of May 16, 2014 had cumulative probabilities of greater than 0.97 with the exception of St. Vincent and the Grenadines, where the probability was 0.65 (range: 0.34–0.87). For some locations (e.g., Buenos Aires and Santiago) monthly risk was generally low, but the cumulative probability of receiving at least one infected traveler over five months was high.

**Figure 2:**
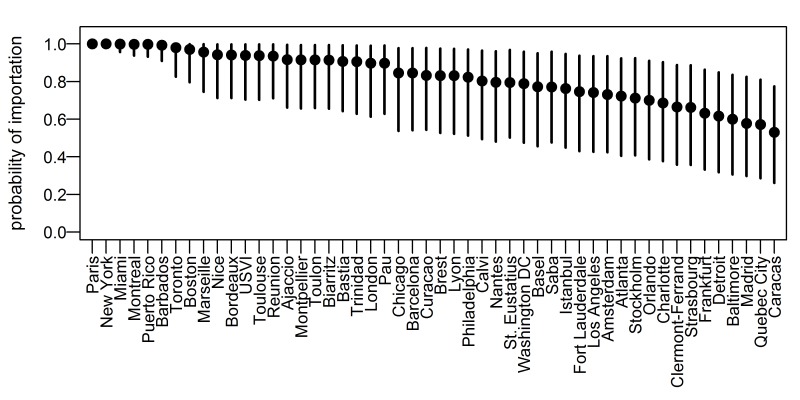
Probability of chikungunya virus importation for select locations, April 2014. Location-specific mean estimates (points) and 95% percentiles (lines) for the predicted probability of the arrival of at least one chikungunya infected traveler for the 50 locations most likely to have had imported cases in April. USVI: U.S. Virgin Islands.

In April, locations with a high probability of importation included those near to current outbreak locations (e.g., Puerto Rico or Barbados), major international cities (e.g., Paris, or New York), and smaller French cities (e.g., Marseille or Nice) (Figure 2, Table S1 in File S1). For areas with very high probabilities of importation, there was little uncertainty in the outcome probability, while for areas with lower probabilities the uncertainty was greater.

### Probability of local transmission in new locations

**Figure 3:**
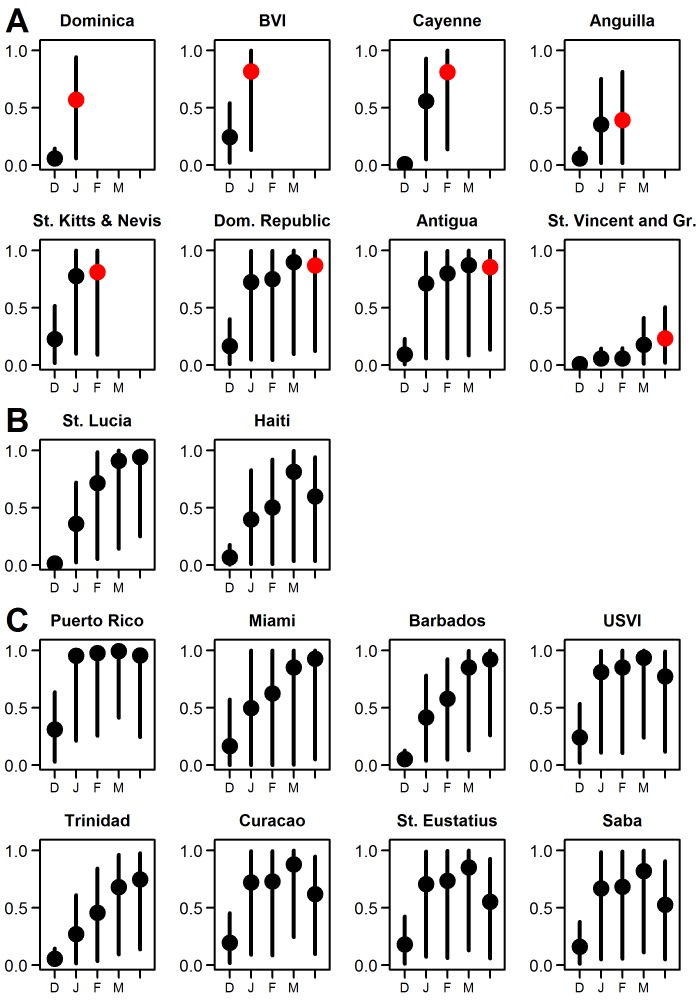
Monthly probabilities of local transmission of chikungunya virus for select locations, December 2013–April 2014. Mean estimates (points) and 95% percentiles (lines) of predictions for the probability of introduced local transmission by month (December (D), January (J), February (F), March (M), and April (A)). **A.** Locations with reported autochthonous cases prior to May 2, 2104. The red points represent the month when cases were first reported. **B.** St. Lucia and Haiti had reported cases in early May. **C.** The eight locations with a predicted probability of local transmission greater than 0.5 in April and no history of cases as of May 16. BVI: British Virgin Islands; USVI: U.S. Virgin Islands.

**Figure 4:**
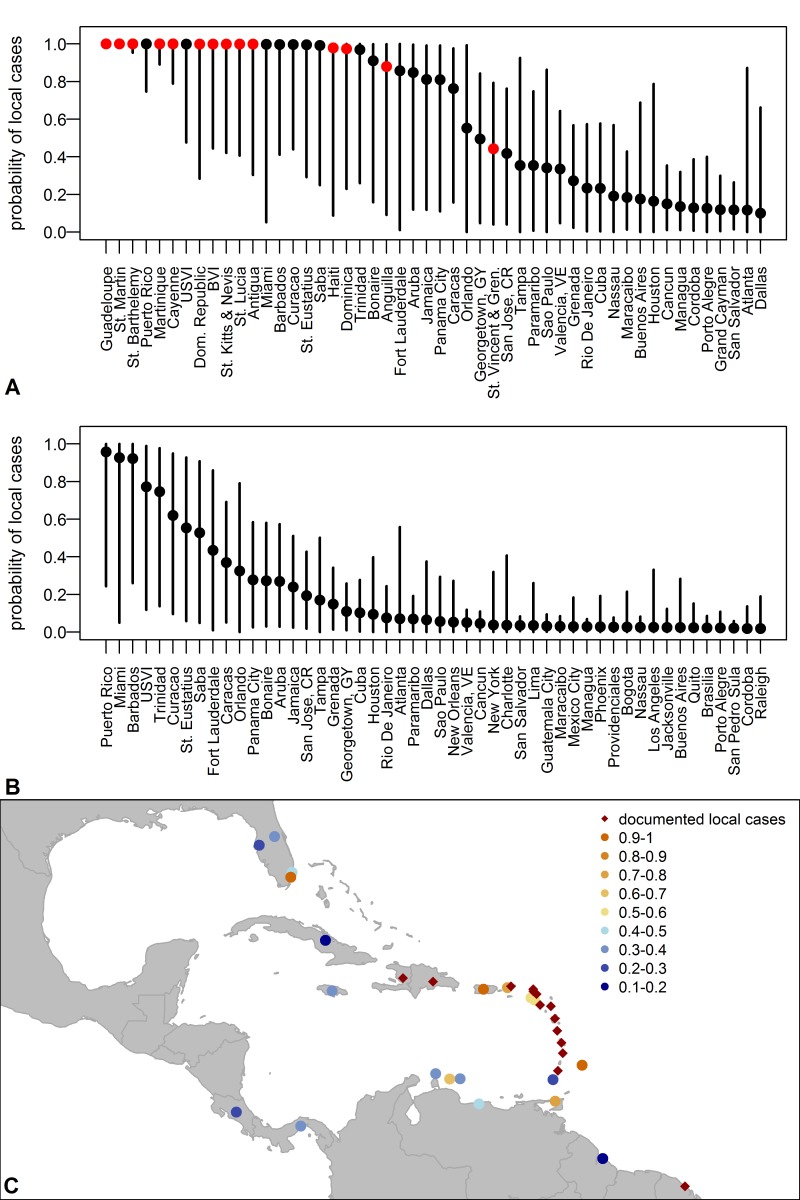
Probability of local transmission of chikungunya virus by location. **A.** Location-specific mean estimates (points) and 95% percentiles (lines) of the cumulative predicted probabilities of local transmission for the 50 locations most likely to have had introduced transmission over the time period December 2013–April 2014. Locations which had reported locally-acquired cases as of May 2 are marked in red. **B.** Location-specific mean estimates (points) and 95% percentiles (lines) of the predicted probabilities for the 50 locations most likely to have had introduced transmission in April. **C.** The mean probabilities of local transmission for all locations in the Americas with p>0.1 in April. BVI: British Virgin Islands; USVI: U.S. Virgin Islands.

The predicted probabilities for introduced transmission generally increased each month as more cases occurred, more locations experienced local cases, and temperatures increased (Figure 3). St. Martin, Guadeloupe, Martinique, and St. Barthelemy all had local cases reported in December. Of the other eight locations with reported local cases in January-April, six had a mean probability of introduced transmission greater than 0.5 in the month when the first case was reported and four of those also had high probabilities in previous months. Anguilla and St. Vincent and the Grenadines had mean monthly probabilities of less than 0.5 (0.25 and 0.23 in April, respectively), but cumulative probabilities close to or above 0.5 (0.88 and 0.44, respectively). The two locations with newly reported local cases in May, St. Lucia and Haiti, had high probabilities of introduced transmission in previous months. Of all the locations predicted to have at least one local case since December 8 of the top 10 had reported cases as of May 16 (Figure 4A).

In April, seven islands in the Caribbean and Miami were probable locations for newly-introduced transmission (Figures 3, 4B–C, Table S2 in File S1). For some locations, the mean predicted probability increased in April while for others the probability decreased, reflecting changes in the numbers and locations of new cases in April and changes in temperatures. For all of these locations there was substantial uncertainty.

## Discussion

The CHIKV outbreak in the Americas that started in December 2013 continues to spread and affect new areas [23]. Being able to identify areas at risk for the introduction and spread of CHIKV in a timely fashion is critical to alerting people to the risk of disease and to implementing control measures. Using branching process models with the current distribution of reported cases, probable travel patterns, and estimated uncertainties we predicted likely locations for introduction and autochthonous transmission of CHIKV. For instance, the models predicted high and increasing probabilities of introduction into St. Lucia and Haiti in recent months and cases were reported from those locations in early May [6]. However, numerous other locations predicted by the models to have infected travelers and introduced transmission had not reported cases as of May 16. These differences between the model predictions and the current epidemiological data may reflect a lack of or delay in recognition of cases, the chance that a probable outcome does not occur, or model error.

Identification and reporting of early cases is challenging and slow as cases must be found, recognized, and confirmed. In many locations clinicians have never seen a chikungunya case and differentiating a rare case from more common dengue cases is difficult. Testing and reporting for chikungunya is also new for most of the affected and at-risk locations. These challenges cause a lag between the observed extent of spread and the true extent of spread, which we predict here. Stochasticity may also contribute to differences between the model and reality. Though an infected traveler or a local infection may be likely in the model and in reality, it is never guaranteed to happen. For St. Vincent and the Grenadines, for example, the mean cumulative probability of having local transmission was 0.44, i.e. similar to flipping a coin.

Uncertainty in the data and parameters leads to uncertainty in the model outcomes, captured here by global sensitivity analysis. These uncertainties had relatively little impact on the probability of infected travelers arriving in locations with high travel flow from affected areas. Thus, even under the most conservative estimates, it is highly likely that there have been unrecognized or unreported cases in travelers in numerous locations. There was much more uncertainty about the probability of local transmission. This is evident in the distribution of *R*_0_, which encompassed *R*_0_ values from other models of CHIKV transmission [36], [37], [38] but also exhibited high variability reflecting uncertainties in key parameters and potentially diverse local mosquito populations. In areas where the risk of introduced transmission is high but uncertain, further work can be done to reduce uncertainty such as characterizing the number of travelers arriving from potential source locations, measuring the actual vector density, or assessing local vector competence.

Despite the limitations and uncertainties, eight of the ten locations predicted to be the most likely locations for introduced transmission by the end of March had documented cases by the end of April. One of these, the Dominican Republic, reported no cases in March and 7,537 in April [22], [23]. This indicates that the model is capturing key characteristics of spread. However, it does not include every nuance of transmission and movement and is intended to provide guidance, not a definitive answer to how the epidemic is currently evolving. For example, the model could not predict spread to Jost Van Dyke (British Virgin Islands), an island with confirmed cases but no airport, where the virus must have arrived via infected travelers on a boat [39]. Nonetheless, the model did predict the likely introduction to the British Virgin Islands.

Rapid assessment of the potential for outbreaks to spread based on limited information is critical to public health planning. Models to assess these risks must, by nature, simplify the complex dynamics of international travel and disease transmission. The models developed here leverage previous work suggesting that a branching process model of spread over a heterogeneous network could capture most of the variability in a more complex stochastic simulation model [14]. One of the fundamental advantages of this approach is that predictions of current spread patterns may be made quickly based solely on knowledge of mobility networks, reported case counts, and coarse characterization of key transmission parameters.

The results presented here indicate that the CHIKV epidemic in the Americas is likely to be expanding, both now and in the future, as more cases occur and temperatures in the Northern Hemisphere increase. In all locations where the probability of imported cases is high, public health authorities should raise awareness in the healthcare community to identify and provide care for cases and to alert travelers to the potential risk of disease and appropriate prevention measures (e.g., use of mosquito repellant) [5], [40]. Additionally, in areas where there is risk for local transmission, public health authorities and partners should begin to plan and consider implementing appropriate interventions (e.g., personal protection against mosquito bites or mosquito control) that could mitigate the risk of local transmission [5]. Implementing public health actions is critical for any outbreak and is ideally informed by careful assessment of risk.

## Supporting Information

File S1 A supporting figure showing the distribution of simulated *R*_0_ values and tables showing the estimated probabilities of imported cases and introduced local transmission by location for the month of April.
(DOCX)
