# Supplementary material for: Nowcasting the Spread of Chikungunya Virus in the Americas
Source: PLoS One. 2014 Aug 11;9(8):e104915. doi: 10.1371/journal.pone.0104915 (PMC4128737; doi:10.1371/journal.pone.0104915)
Supplement: File S1 — A supporting figure showing the distribution of simulated R 0 values and tables showing the estimated probabilities of imported cases and introduced local transmission by location for the month of April. (DOCX) [file pone.0104915.s001.docx]

**Supporting Information Contents**

**Figure S1. p. 2**

Distribution of *R*_0_ from sampled parameters.

**Table S1. p. 3**

Probability of chikungunya virus importation by location, April 2014.

**Table S2. p. 5**

Probability of local transmission of chikungunya virus by location in the Americas, April 2014.


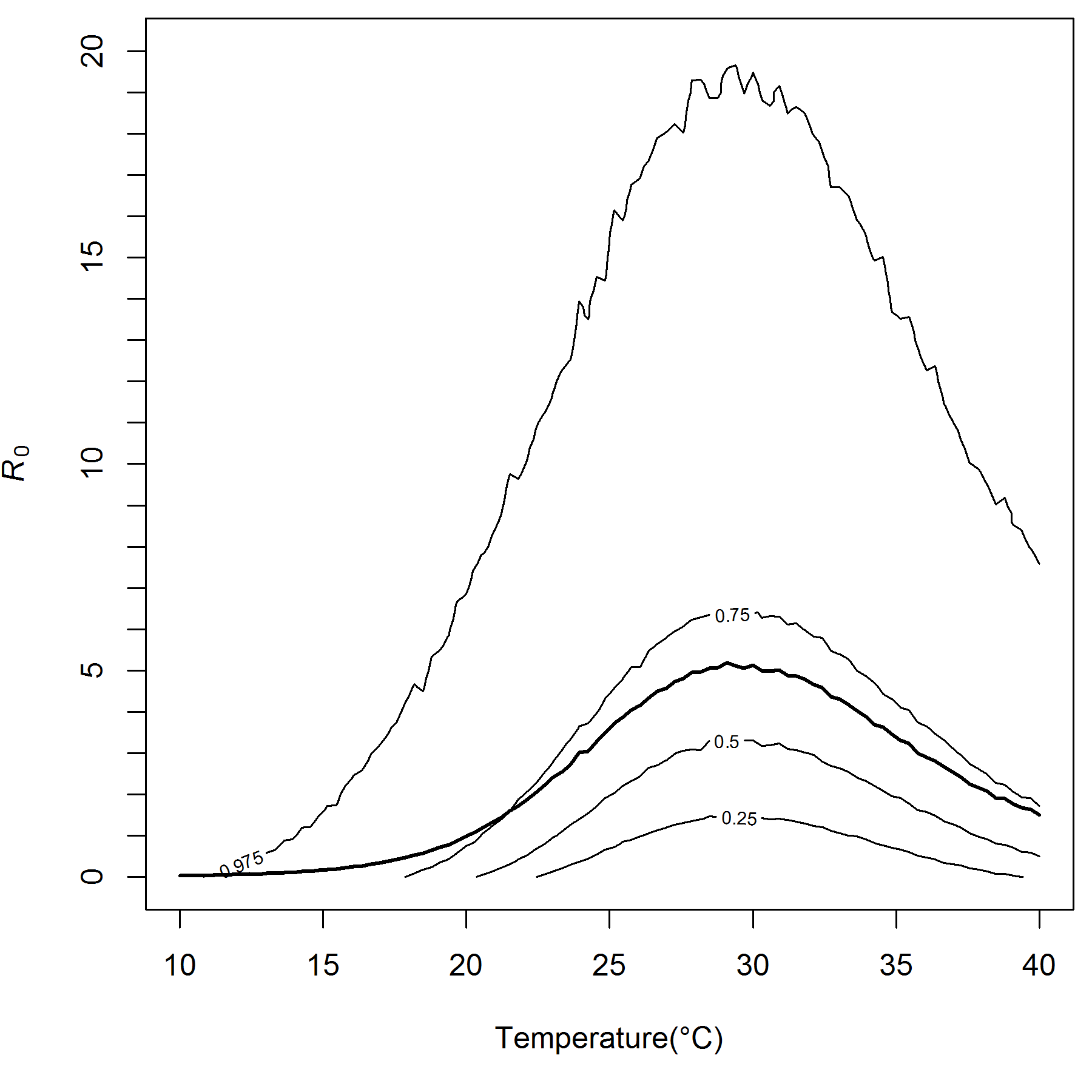


**Figure S1. Distribution of *R*_0_ from sampled parameters.** For each of 10,000 sampled sets of model parameters, we calculated the *R*_0_ value at different temperatures ($R_{0}=R_{0}^{HM}R_{0}^{MH}$). The thick line is the mean of these samples and the thin lines are the 25^th^, 50^th^, 75^th^, and 97.5^th^ percentiles of that distribution.

**Table S1. Probability of chikungunya virus importation by location, April 2014.***

|  | mean | | (range) |  |  | mean | (range) |
| --- | --- | --- | --- | --- | --- | --- | --- |
| Paris | 1.00 | | (1.00-1.00) |  | Cleveland | 0.36 | (0.16-0.57) |
| New York | 1.00 | | (1.00-1.00) |  | Buffalo | 0.33 | (0.15-0.54) |
| Miami | 1.00 | | (0.96-1.00) |  | Berlin | 0.32 | (0.14-0.53) |
| Montreal | 1.00 | | (0.94-1.00) |  | Lima | 0.32 | (0.14-0.53) |
| Puerto Rico | 1.00 | | (0.93-1.00) |  | Raleigh | 0.32 | (0.14-0.52) |
| Barbados | 0.99 | | (0.91-1.00) |  | San Jose, CR | 0.31 | (0.14-0.51) |
| Toronto | 0.98 | | (0.83-1.00) |  | Sao Paulo | 0.30 | (0.13-0.50) |
| Boston | 0.97 | | (0.80-1.00) |  | Lisbon | 0.28 | (0.12-0.48) |
| Marseille | 0.96 | | (0.75-1.00) |  | St. Louis | 0.28 | (0.12-0.47) |
| Nice | 0.94 | | (0.71-1.00) |  | Mauritius | 0.28 | (0.12-0.47) |
| Bordeaux | 0.94 | | (0.71-1.00) |  | New Orleans | 0.27 | (0.12-0.46) |
| USVI | 0.94 | | (0.70-1.00) |  | Pittsburgh | 0.27 | (0.12-0.46) |
| Toulouse | 0.94 | | (0.70-1.00) |  | Stuttgart | 0.27 | (0.12-0.45) |
| Reunion | 0.94 | | (0.71-1.00) |  | Seattle | 0.27 | (0.11-0.45) |
| Ajaccio | 0.92 | | (0.66-1.00) |  | Bogota | 0.26 | (0.11-0.45) |
| Montpellier | 0.92 | | (0.66-0.99) |  | Columbus | 0.26 | (0.11-0.44) |
| Toulon | 0.92 | | (0.66-1.00) |  | Grenada | 0.25 | (0.10-0.42) |
| Biarritz | 0.91 | | (0.66-0.99) |  | Edmonton | 0.24 | (0.10-0.42) |
| Bastia | 0.91 | | (0.64-0.99) |  | Ottawa | 0.24 | (0.10-0.41) |
| Trinidad | 0.91 | | (0.63-0.99) |  | Calgary | 0.24 | (0.10-0.41) |
| London | 0.90 | | (0.61-0.99) |  | Cincinnati | 0.24 | (0.10-0.40) |
| Pau | 0.90 | | (0.63-0.99) |  | Brussels | 0.23 | (0.10-0.40) |
| Chicago | 0.85 | | (0.54-0.98) |  | Oslo | 0.23 | (0.10-0.40) |
| Barcelona | 0.85 | | (0.54-0.98) |  | Milwaukee | 0.23 | (0.10-0.40) |
| Curacao | 0.83 | | (0.54-0.98) |  | Warsaw | 0.23 | (0.10-0.40) |
| Brest | 0.83 | | (0.53-0.98) |  | Valencia | 0.23 | (0.10-0.39) |
| Lyon | 0.83 | | (0.52-0.97) |  | Phoenix | 0.22 | (0.09-0.37) |
| Philadelphia | 0.82 | | (0.51-0.97) |  | Palma de Mallorca | 0.21 | (0.09-0.37) |
| Calvi | 0.80 | | (0.49-0.97) |  | Indianapolis | 0.21 | (0.09-0.37) |
| Nantes | 0.80 | | (0.48-0.96) |  | Cuba | 0.21 | (0.09-0.36) |
| St. Eustatius | 0.79 | | (0.50-0.97) |  | Palermo | 0.20 | (0.09-0.36) |
| Washington DC | 0.79 | | (0.48-0.96) |  | Rochester | 0.20 | (0.08-0.35) |
| Basel | 0.77 | | (0.46-0.95) |  | Mexico City | 0.20 | (0.08-0.35) |
| Saba | 0.77 | | (0.48-0.96) |  | Portland | 0.20 | (0.08-0.35) |
| Istanbul | 0.76 | | (0.45-0.95) |  | Syracuse | 0.20 | (0.08-0.35) |
| Fort Lauderdale | 0.75 | | (0.43-0.94) |  | Perpignan | 0.20 | (0.08-0.35) |
| Los Angeles | 0.74 | | (0.43-0.94) |  | Richmond | 0.20 | (0.08-0.34) |
| Amsterdam | 0.73 | | (0.42-0.93) |  | Rio De Janeiro | 0.20 | (0.08-0.34) |
| Atlanta | 0.72 | | (0.41-0.92) |  | Manchester | 0.20 | (0.08-0.34) |
| Stockholm | 0.71 | | (0.41-0.93) |  | Las Vegas | 0.19 | (0.08-0.33) |
| Orlando | 0.70 | | (0.39-0.91) |  | Oran | 0.19 | (0.08-0.33) |
| Charlotte | 0.69 | | (0.38-0.90) |  | Georgetown, GY | 0.18 | (0.08-0.32) |
| Clermont-Ferrand | 0.66 | | (0.36-0.89) |  | Vancouver | 0.18 | (0.07-0.31) |
| Strasbourg | 0.66 | | (0.36-0.89) |  | Quito | 0.17 | (0.07-0.30) |
| Frankfurt | 0.63 | | (0.33-0.86) |  | Providence | 0.17 | (0.07-0.30) |
| Detroit | 0.62 | | (0.32-0.85) |  | Quimper | 0.17 | (0.07-0.29) |
| Baltimore | 0.60 | | (0.31-0.84) |  | Prague | 0.16 | (0.06-0.28) |
| Madrid | 0.58 | | (0.30-0.83) |  | Cordoba | 0.16 | (0.06-0.28) |
| Quebec City | | 0.57 | (0.29-0.81) |  | Lourdes | 0.15 | (0.06-0.27) |
| Caracas | | 0.53 | (0.26-0.78) |  | Rennes | 0.15 | (0.06-0.26) |
| Geneva | | 0.53 | (0.25-0.76) |  | Paramaribo | 0.15 | (0.06-0.26) |
| Moscow | | 0.50 | (0.25-0.75) |  | St. Petersburg | 0.15 | (0.06-0.26) |
| Milan | | 0.50 | (0.24-0.74) |  | Luxembourg | 0.14 | (0.06-0.25) |
| Buenos Aires | | 0.49 | (0.24-0.74) |  | Norfolk | 0.14 | (0.06-0.25) |
| Duesseldorf | | 0.49 | (0.23-0.73) |  | Bilbao | 0.14 | (0.06-0.24) |
| Hartford | | 0.49 | (0.24-0.74) |  | Lorient | 0.13 | (0.05-0.24) |
| Munich | | 0.49 | (0.23-0.73) |  | Bejaia | 0.13 | (0.05-0.24) |
| Rome | | 0.48 | (0.23-0.72) |  | Ibiza | 0.13 | (0.05-0.24) |
| Minneapolis | | 0.48 | (0.23-0.72) |  | Salt Lake City | 0.13 | (0.05-0.24) |
| Algiers | | 0.47 | (0.22-0.71) |  | Setif | 0.13 | (0.05-0.23) |
| Copenhagen | | 0.46 | (0.21-0.69) |  | Kansas City | 0.12 | (0.05-0.22) |
| Halifax | | 0.45 | (0.22-0.70) |  | Belo Horizonte | 0.12 | (0.05-0.22) |
| Zurich | | 0.45 | (0.21-0.69) |  | Jacksonville | 0.12 | (0.05-0.21) |
| San Francisco | | 0.45 | (0.21-0.68) |  | Nashville | 0.12 | (0.05-0.21) |
| Panama City | | 0.44 | (0.21-0.68) |  | Louisville | 0.12 | (0.05-0.21) |
| Denver | | 0.43 | (0.20-0.67) |  | San Diego | 0.11 | (0.05-0.21) |
| Tampa | | 0.43 | (0.20-0.66) |  | Alicante | 0.11 | (0.05-0.21) |
| Bonaire | | 0.43 | (0.20-0.67) |  | Bologna | 0.11 | (0.04-0.20) |
| Santiago | | 0.42 | (0.19-0.66) |  | Hong Kong | 0.11 | (0.04-0.20) |
| Dallas | | 0.41 | (0.19-0.64) |  | Lille | 0.11 | (0.04-0.20) |
| Vienna | | 0.41 | (0.19-0.64) |  | Dublin | 0.11 | (0.04-0.20) |
| Hamburg | | 0.40 | (0.18-0.63) |  | Albany | 0.11 | (0.04-0.19) |
| Houston | | 0.38 | (0.17-0.60) |  | Beijing | 0.10 | (0.04-0.19) |
| Jamaica | | 0.38 | (0.17-0.60) |  | Porto Alegre | 0.10 | (0.04-0.19) |
| Budapest | | 0.37 | (0.17-0.60) |  | Burlington | 0.10 | (0.04-0.19) |
| Tunis | | 0.37 | (0.17-0.59) |  | Lalle | 0.10 | (0.04-0.19) |

*For all locations with mean *p* > 0.10.

**Table S2. Probability of local transmission of chikungunya virus by location in the Americas, April 2014.***

|  | mean | (range) |
| --- | --- | --- |
| Puerto Rico | 0.96 | (0.24-1.00) |
| Miami | 0.93 | (0.05-1.00) |
| Barbados | 0.92 | (0.26-1.00) |
| USVI | 0.77 | (0.12-0.99) |
| Trinidad | 0.75 | (0.14-0.98) |
| Curacao | 0.62 | (0.10-0.95) |
| St. Eustatius | 0.55 | (0.06-0.93) |
| Saba | 0.53 | (0.05-0.91) |
| Fort Lauderdale | 0.43 | (0.01-0.86) |
| Caracas | 0.37 | (0.05-0.69) |
| Orlando | 0.32 | (0.00-0.79) |
| Panama City | 0.28 | (0.03-0.59) |
| Bonaire | 0.27 | (0.03-0.58) |
| Aruba | 0.27 | (0.03-0.57) |
| Jamaica | 0.24 | (0.02-0.51) |
| San Jose, CR | 0.19 | (0.02-0.43) |
| Tampa | 0.17 | (0.00-0.50) |
| Grenada | 0.15 | (0.01-0.34) |
| Georgetown, GY | 0.11 | (0.01-0.26) |
| Cuba | 0.10 | (0.00-0.28) |

*For all locations with mean *p* > 0.10.
